# DeepTag: inferring diagnoses from veterinary clinical notes

**DOI:** 10.1038/s41746-018-0067-8

**Published:** 2018-10-24

**Authors:** Allen Nie, Ashley Zehnder, Rodney L. Page, Yuhui Zhang, Arturo Lopez Pineda, Manuel A. Rivas, Carlos D. Bustamante, James Zou

**Affiliations:** 1 Department of Biomedical Data Science, Stanford University, Stanford, CA 94305 USA; 2 Department of Clinical Sciences, Colorado State University, Fort Collins, CO 80523 USA; 3 Department of Computer Science and Technology, Tsinghua University, Beijing, China; 4 Chan-Zuckerberg Biohub, San Francisco, CA 94158 USA

**Keywords:** Public health, Health services

## Abstract

Large scale veterinary clinical records can become a powerful resource for patient care and research. However, clinicians lack the time and resource to annotate patient records with standard medical diagnostic codes and most veterinary visits are captured in free-text notes. The lack of standard coding makes it challenging to use the clinical data to improve patient care. It is also a major impediment to cross-species translational research, which relies on the ability to accurately identify patient cohorts with specific diagnostic criteria in humans and animals. In order to reduce the coding burden for veterinary clinical practice and aid translational research, we have developed a deep learning algorithm, DeepTag, which automatically infers diagnostic codes from veterinary free-text notes. DeepTag is trained on a newly curated dataset of 112,558 veterinary notes manually annotated by experts. DeepTag extends multitask LSTM with an improved hierarchical objective that captures the semantic structures between diseases. To foster human-machine collaboration, DeepTag also learns to abstain in examples when it is uncertain and defers them to human experts, resulting in improved performance. DeepTag accurately infers disease codes from free-text even in challenging cross-hospital settings where the text comes from different clinical settings than the ones used for training. It enables automated disease annotation across a broad range of clinical diagnoses with minimal preprocessing. The technical framework in this work can be applied in other medical domains that currently lack medical coding resources.

## Introduction

While a robust medical coding infrastructure exists in the US healthcare system for human medical records, this is not the case in veterinary medicine, which lacks coding infrastructure and standardized nomenclatures across medical institutions. Most veterinary clinical notes are not coded with standard SNOMED-CT diagnosis.^1^ This hampers efforts at clinical research and public health monitoring. Due to the relative ease of obtaining large volumes of free-text veterinary clinical records for research (compared to similar volumes of human medical data) and the importance of turning these volumes of text into structured data to advance clinical research, we investigated effective methods for building automatic coding systems for the veterinary records.

It is becoming increasingly accepted that spontaneous diseases in animals have important translational impact on the study of human disease for a variety of disciplines.^2^ Beyond the study of zoonotic diseases, which represent 60–70% of all emerging diseases, noninfectious diseases, like cancer, have become increasingly studied in companion animals as a way to mitigate some of the problems with rodent models of disease.^3^ Additionally, spontaneous models of disease in companion animals are being used in drug development pipelines as these models more closely resemble the “real world” clinical settings of diseases than genetically altered mouse models.^4–7^ However, when it comes to identifying clinical cohorts of veterinary patients on a large scale for clinical research, there are several problems. One of the first is that veterinary clinical visits rarely have diagnostic codes applied to them, either by clinicians or medical coders. There is no substantial third party payer system and no HealthIT act that applies to veterinary medicine, so there are few incentives for clinicians or hospitals to annotate their records for diseases to be able to identify patients by diagnosis. Billing codes are largely institution-specific and rarely applicable across institutions, unless hospitals are under the same management structure and records system. Some large corporate practice groups have their own internal clinical coding structures, but that data is rarely made available for outside researchers. A small number (<5) academic veterinary centers (of a total of 30 veterinary schools in the US) employ dedicated medical coding staff that apply disease codes to clinical records so these records can be identified for clinical faculty for research purposes. How best to utilize this rare, well-annotated, veterinary clinical data for the development of tools that can help organize the remaining seqments of the veterinary medical domain is an open area of research.

In this paper, we develop DeepTag, a system to automatically code veterinary clinical notes. DeepTag takes free-form veterinary note as input and infers clinical diagnosis from the note. The inferred diagnosis is in the form of 42 SNOMED-CT codes. We trained DeepTag on a large set of 112,558 veterinary notes, and each note is expert labeled with a set of SNOMED-CT codes. DeepTag is a bidirectional long–short-term memory network (BLSTM) augmented with a hierarchical training objective that captures similarities between the diagnosis codes. We evaluated DeepTag’s performance on challenging cross-hospital coding tasks.

Natural language processing (NLP) techniques have improved from leveraging discrete patterns such as n-grams^8^ to continuous learning algorithms like LSTMs.^9^ This strategy has proven to be very successful when a sizable amount of data can be acquired. Combined with advances in optimization and classification algorithms, the field has developed algorithms that can match or exceed human performance in several traditionally difficult tasks.^10^

Analyzing free text such as diagnostic reports and clinical notes has been a central focus of clinical natural language processing.^11^ Most of the previous research has focused on the human healthcare systems. Examples include using NLP tools to improve pneumonia screening in the emergency department, assisting in adenoma detection, assisting and simplifying hospital processes by identifying billing codes from clinical notes.^12^ In an unsupervised setting, Pivovarov et al.^13^ have conducted experiments to discover phenotypes and diseases on a broad set of heterogeneous data.

In the domain of veterinary medicine, millions of clinical summaries are stored as electronic health records (EHR) in various hospitals and clinics. Unlike human discharge summaries that have been assigned with billing codes (ICD-9/ICD-10 codes), veterinary summaries exist primarily as free text. This makes it challenging to perform systematic analysis such as disease prevalence studies, analysis of adverse drug effects, therapeutic efficacy or outcome analysis. Veterinary domain is very favorable for an NLP system that can convert large amount of free-text notes into structured information. Such a system would benefit the veterinary community in a substantial way and can be deployed in multiple clinical settings. Veterinary medicine is a domain where clinical NLP tools can have a substantial impact in practice and be integrated into daily use.

Identifying a set of conditions/diseases from clinical notes has been actively studied.^12,14^ Currently, the task of transforming free text into structured information primarily relies on two approaches: named entity recognition (NER) and automated coding. DeepTag is designed to perform automated coding rather than NER. NER requires annotation on the word level, where each word is associated with one of a few types. In the ShARe task,^15^ the importance is placed on identifying disease span and then normalizing into standard terminology in SNOMED-CT or Unified Medical Language System. In other works, the focus has been on tagging each word with a specific type: adverse drug effect, severity, drug name, etc.^16^ Annotating on word level is expensive, and most corpora contain only a couple of hundreds or thousands of clinical notes. Even though early shared task in this domain has proven to be successful,^17,18^ it is still difficult to curate a large dataset in this manner.

Automated coding on the other hand takes the entire free text as input, and infers a set of codes that are used to code the entire work. Most discharge summaries in human hospitals have billing codes assigned. Baumel et al.^19^ proposed a text processing model for automated coding that processes each sentence first and then processes the encoded hidden states for the entire document. This multilevel approach is especially suitable for longer texts, and the method was applied to the MIMIC data, where each document is on average five times longer than the veterinary notes from Colorodo State University of Veterinary Medicine and Biomedical Sciences (CSU). Rajkomar et al.^20^ used deep learning methods to process the entire EHR and make clinical predictions for a wide range of problems including automated coding. In their work, they compared three deep learning models: LSTM, time-aware feedforward neural network, and boosted time-based decision stumps. In this work, we use a new hierarchical training objective which is designed to capture the similarities among the SNOMED-CT codes. This hierarchical objective is complementary to these previous approaches in the sense that the hierarchical objective can be used on top of any architecture. Our cross-hospital evaluations also extend what is typically done in literature. Even though Rajkomar et al. had data from two hospitals, they did not investigate the performance of the model when trained on one hospital but evaluated on the other. In our work, due to the lack of coded clinical notes in the veterinary community beyond a few academic hospitals, it is especially salient for us to evaluate the model's^™^ ability to generalize across hospitals.

Our work is also related to the work of Kavuluru et al.,^21^ who experimented with different training strategies and compared which strategy is the best for automated coding, and Subotin et al.,^22^ who improved upon direct label probability estimation and used a conditional probability estimator to fine-tune the label probability. Perotte et al.^23^ also investigated possible methods to leverage the hierarchical structure of disease codes by using an support vector machine (SVM) algorithm on each level of the ICD-9 hierarchy tree.

**Fig. 1:**
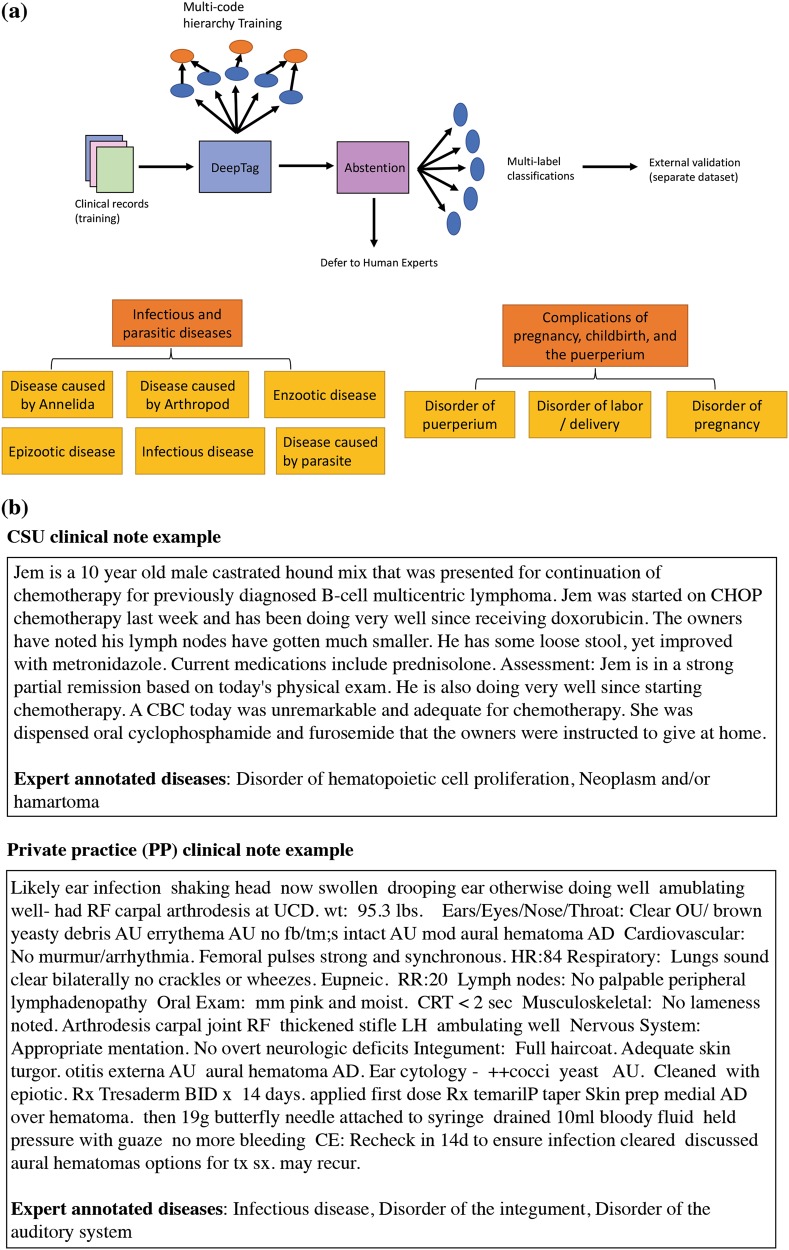
System workflow and clinical note examples. Figure **a** shows the workflow of DeepTag with abstention. Then we show two example meta-diseases corresponding to two subsets of the 42 SNOMED-CT codes. Figure **b** shows two example notes from the CSU and PP datasets

Cross-hospital generalization is a significant challenge in the veterinary coding setting. Most veterinary clinics currently do not apply diagnosis codes to their notes.^1^ Therefore our training data can only come from a handful of university-based regional referral centers that manually code their free-text notes. The task is to train a model on such data and deploy for thousands of private hospitals and clinics. University-based centers and private hospitals and clinics have substantial variation in the writing style, the patient population, and the distribution of diseases (Fig. 1). For example, the training dataset we have used in this work comes from a university-based hospital with a high-volume referral oncology service, but typical local hospitals might face more dermatologic or gastrointestinal cases.

## Results

DeepTag takes clinician’s notes as input and predicts a set of SNOMED-CT disease codes. SNOMED-CT is a comprehensive and precise clinical health terminology managed by the International Health Terminology Standards Development Organization. DeepTag is a BLSTM neural network with a new hierarchical learning objective designed to capture similarities between the disease codes (see the Supplementary for model details).

DeepTag is trained on 112,558 annotated veterinary notes from the CSU curated for research purposes. Each of these notes is a free-text description of a patient visit, and is manually tagged with at least one, and on average 8, out of the 41 SNOMED-CT disease codes by experts. In addition, we map every nondisease related code to an extra code. In total, DeepTag learns to tag a clinical note with a subset of 42 codes.

We evaluate DeepTag on two different datasets. One consists of 5628 randomly sampled nonoverlapping held-out documents from the same CSU dataset that the system is trained on. The other dataset contains 586 documents and are collected from a private practice (PP) located in northern California. Each of the these document is also manually annotated with the appropriate SNOMED-CT codes by human experts. We refer to this dataset as the PP dataset.

We regard the PP dataset as a “out-of-domain” dataset due to its substantial difference with regard to writing style and institution type compared to the CSU dataset.^24^ The PP documents tend to be substantially shorter (average of 253 words compared to 368 words in CSU), use more abbreviations and have different distribution of diseases (see Methods section for more details).

### Tagging performance

We present DeepTag’s performance on the CSU and PP test data in Table 1. To save space, we display the 20 most frequent disease codes in Table 1. For each disease code, we report the number of training examples for the disease code (*N*), the scores for precision, recall, *F*_1_, ROC AUC, and the number of subtypes in this disease code. While DeepTag achieves reasonable *F*_1_ scores overall, its performance is quite heterogeneous for different disease codes. Moreover, the performance decreases when DeepTag is applied to the out-of-domain PP test data. We identify two factors that substantially impact DeepTag’s performance: (1) the number of training examples that are tagged with the given disease code and (2) the number of subtypes, where a subtype is a SNOMED-CT code applied to either dataset that is lower in the SNOMED-CT hierarchy than the top-level disease codes DeepTag is predicting. We use the number of subtypes as a proxy for the diversity and specificity of the clinical text descriptions. Thus, a higher number of subtypes suggests a wider spectrum of diseases.

**Table 1:** Report of DeepTag performance on the CSU test data and PP data

	CSU						PP (Cross-hospital)					
Disease code	*N*	Prec	Rec	*F*_1_	AUC	Sub	*N*	Prec	Rec	*F*_1_	AUC	Sub
Autoimmune disease	1280	94	72.3	81.4	0.86	11	1	0	0	0	0.5	1(1)
Congenital disease	3345	72.9	35.9	47.3	0.68	224	17	46.7	3.5	6.4	0.52	8(6)
Propensity to adverse reactions	5105	89.1	70.2	78.1	0.85	8	43	67.2	12.6	19.5	0.56	7(2)
Metabolic disease	5265	68.9	55.4	61	0.77	82	26	56.6	48.5	51.1	0.73	12(9)
Disorder of auditory system	5393	81	66.2	72.8	0.83	67	64	78.8	70.3	73.8	0.84	12(6)
Hypersensitivity condition	6871	85.7	74.6	79.5	0.87	31	50	67.7	22.4	31.6	0.61	11(4)
Disorder of endocrine system	7009	79.2	66.7	72.2	0.83	84	46	44.4	21.7	28.7	0.6	8(8)
Disorder of hematopoietic cell proliferation	7294	95.1	87.4	91	0.94	22	16	62.7	25	34.5	0.62	6(1)
Disorder of nervous system	7488	76.1	63.8	69.2	0.81	243	27	40.4	26.7	30.8	0.62	19(14)
Disorder of cardiovascular system	8733	79.3	62.5	69.7	0.81	351	53	44.1	52.1	46.4	0.73	30(24)
Disorder of the genitourinary system	8892	77.7	62.6	69.3	0.81	317	44	47.8	39.1	42.2	0.68	19(12)
Traumatic AND/OR nontraumatic injury	9027	72.8	57.2	63.5	0.78	536	19	50.5	15.8	23.1	0.58	13(8)
Visual system disorder	10139	84.3	81.1	82.6	0.9	413	62	65	62.6	63.2	0.79	39(34)
Infectious disease	11304	71.2	53.7	60.8	0.76	260	88	63.8	23	32.3	0.6	20(10)
Disorder of respiratory system	11322	79.5	65.5	71.8	0.82	274	27	38.3	42.2	38.2	0.69	16(14)
Disorder of connective tissue	17477	75.4	67	70.7	0.81	567	24	30.4	24.2	26.3	0.61	15(11)
Disorder of musculoskeletal system	20060	77	73.4	74.8	0.84	670	56	54	41.4	46.1	0.69	31(19)
Disorder of integument	21052	84.2	71.6	77.3	0.84	360	156	65.7	60.1	62.6	0.74	58(32)
Disorder of digestive system	22589	76.8	67.1	71.5	0.81	694	195	58	47.9	51.3	0.65	47(36)
Neoplasm and/or hamartoma	36108	92.2	88.9	90.5	0.93	749	59	26.1	72.5	37.8	0.74	18(7)

This table reports the DeepTag’s performance (precision, recall, *F*_1_ and AUC) for the 20 most frequent disease codes (from a total of 42 disease codes). *N* indicates the total number of examples in the dataset. AUC refers to area under the receiver operator curve. Sub indicates the number of lower-level disease codes that are present in the dataset that are binned into one of the disease level codes. For the PP dataset, the Sub number in parentheses indicate the number of subtypes that are also present in CSU dataset.

#### Performance improves with more training examples

We first note that DeepTag works relatively well when the number of training examples for each disease code is abundant. We generate a scatter plot to capture the correlation between number of examples in the in CSU dataset and the disease code’s *F*_1_ score evaluated on the CSU test set. We also plot the *F*_1_ score for the disease code evaluated on the PP dataset and its number of training examples on the CSU dataset.

**Fig. 2:**
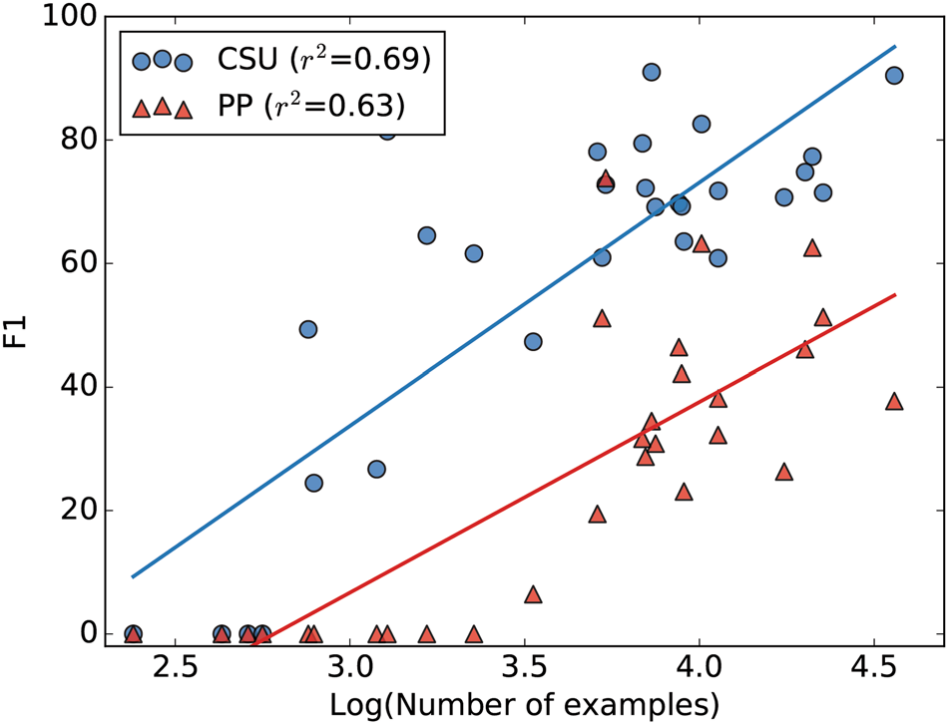
Per-disease code *F*_1_ score plotted with log of number of examples in the training dataset. Results shown here are from the DeepTag model. Each point represents a disease code, its corresponding number of training examples in CSU, and the per-disease code *F*_1_ score from the DeepTag model

For the CSU dataset, we observe an almost linear relationship between the log number of examples and the *F*_1_ score in Fig. 2, suggesting that our large training data is crucial for the prediction performance. We observe a similar pattern when evaluating on the PP dataset, thought the correlation is weaker and the pattern is less linear. This is due to the out-of-domain nature of PP, which we investigate in depth below.

#### More diverse disease codes are harder to predict

After observing the general correlation between number of training examples and per-disease code *F*_1_ scores, we can investigate outliers. These are diseases that have many examples but on which DeepTag performed poorly and diseases that have few examples but DeepTag performed well. For *disorder of digestive system*, despite having the second highest number of training examples (22,589), both precision and recall are lower than other frequent diseases. We find that this disease code covers the second largest number of subtypes (694). On the other hand, *disorder of hematopoietic cell proliferation* has the highest *F*_1_ score with relatively few training examples (*N* = 7294). This disease code has only 22 subtypes. Similarly *autoimmune diseases* has few training examples (*N* = 1280) but it still has a relatively high *F*_1_, and it also has only 11 subtypes.

The number of subtypes—i.e., the number of different lower-level SNOMED-CT codes that are mapped to each of the 42 higher-level disease code—can serve as an indicator for the diversity or specificity of the text descriptions. For a disease like *disorder of digestive system*, it subsumes many different types of diseases such as *periodontal disease*, *hepatic disease*, and *disease of stomach*, which all have different diagnoses. Similarly, *neoplasm and/or hamartoma* encapsulates many different histologic types and be categorized as benign, malignant, or unknown, thus resulting in many different lower-level codes (749 codes) being mapped into the same top-level disease code. DeepTag needs to associate diverse descriptions to the same high-level disease code, increasing the difficulty of the prediction task.

We hypothesize that disease codes with many subtypes will be difficult for the system to predict. This hypothesis suggests that the number of subtypes a disease code contains could explain some of the heterogeneity in DeepTag’s performance beyond the heterogeneity due to the training sample size.

We conduct a multiple linear regression test with both the number of training examples as well as number of subtypes each disease code contains as covariates and the *F*_1_ score as the outcome. In the regression, the coefficient for number of subtypes is negative with *p* < 0.001. This indicates that, controlling for the number of training examples, having more subtypes in a disease code makes tagging more challenging and decreases DeepTag’s performance on the disease code.

#### Performance on PP

Next we investigate DeepTag’s performance discrepancy between the CSU and PP test data. A primary contributing factor to the discrepancy is that the underlying text in PP is stylistically and functionally different from the text in CSU. Note that DeepTag was only trained on the CSU text and was not fine-tuned on PP. The example texts in Fig. 1 illustrate the striking difference. In particular, PP uses many more abbreviations that are not observed in CSU.

After filtering out numbers, 15.4% of words in PP are not found in CSU. Many of the PP specific words appear to be medical acronyms that are not used in CSU or terms that describe test results or medical procedures. Since these vocabulary has no trained word embedding from the CSU dataset, DeepTag can not leverage them in the disease tagging process.

Despite having many training examples, DeepTag performs poorly on some very frequent diseases, for example, *neoplasm and/or hamartoma*. On the opposite end of the spectrum, the tagger does well for *disorder of auditory system* on both CSU and PP dataset, despite only having a moderate amount of training examples. Besides the main issue of vocabulary mismatch, many subtypes (lower-level disease codes) that get mapped to a disease code in CSU do not exist in PP, and subtypes in PP also might not exist in CSU. We refer to this as the subtype distribution shift. For example, In CSU, *neoplasm and/or hamartoma* has 749 observed subtypes. Only 7 out of 749 subtypes are present in PP. Moreover, there are 11 subtypes are unique to the PP dataset and are not observed in the CSU training set.

In addition to the subtype analysis, we note that for rarer diseases, the precision drop between CSU and PP is not as deep as the recall drop. This can be interpreted as the model is fairly confident and precise about the key phrases it discovered from the CSU dataset. The drop in recall in the PP dataset could be partially due the fact that PP uses many terms and phrases that are not in the CSU data.

### Improvements from disease similarity

The 42 disease codes can be naturally grouped into 18 meta-diseases; each meta-disease corresponds to a subset of diseases that are related to each other (see the Supplementary). For example, the disease codes for “Disease caused by Arthropod” and “Disease caused by Annelida” belong to the same meta-disease: “Infectious and parasitic diseases”. We designed DeepTag to leverage this hierarchical structure amongst the disease codes. Intuitively, suppose the true disease associated with a note is *A* and DeepTag mistakenly predicts disease code *B*. Then its penalty should be larger if *B* is very different from *A*—i.e., they are in different meta-diseases—than if *B* and *A* are in the same meta-disease. More precisely, we use the grouping of similar codes into meta-diseases as a regularization in the training objective of DeepTag. Basic deep learning systems like LSTM and BLSTM do not incorporate this information.

DeepTag uses a *L*_2_-based distance objective to place this constraint between disease code embeddings, which are the parameters in the final layer of the DeepTag neural network. The objective encourages the embeddings of disease codes that are in the same meta-disease to be closer to each other than the embeddings of disease codes across different meta-diseases. In addition, we investigated another approach that can also leverage disease similarity: DeepTag-M. This method computes the probability of a meta-disease based on the probability of the disease codes that are grouped into it. Instead of forcing similarity constraints on disease code embeddings, DeepTag-M encourages the model to make correct prediction on the meta-diseases as well as on the disease codes (see the Supplementary).

In Table 2, we compare the performance of DeepTag and DeepTag-M with the standard LSTM, BLSTM, text convolutional neural network (CNN), MetaMap-SVM, and MetaMap-MLP. MetaMap is a nondeep learning approach that processes each document to extract a set of medically relevant terms.^25^ We then train a SVM (MetaMap-SVM) or a multilayer perceptron (MetaMap-MLP) to use the extracted terms to predict disease codes.

**Table 2:** Evaluation of trained classifiers on the CSU and PP data

Model	EM	Precision		Recall		*F*_1_	
		unwgt	wgt	unwgt	wgt	unwgt	wgt
*CSU data*							
MetaMap-SVM	32.2	52.2	74.8	54.8	75	53.2	74.8
MetaMap-MLP	41.2	64.7	82.6	48.5	71.8	55	76.4
CNN	45.1	73.1	84.4	57.2	78.4	62.2	80.9
LSTM	47.4	76.6	85.9	59.3	78.7	65.3	81.7
BLSTM	48.2	76.1	86	57.6	79.4	63.5	82.2
DeepTag-M	**48.6**	76.8	**86.3**	58.7	79.6	64.6	82.4
DeepTag	48.4	**79.9**	86.1	**62.1**	**79.8**	**68**	**82.4**
*PP data (cross-hospital)*							
MetaMap-SVM	3.2	26.5	57.3	37.7	53.1	24.8	51.6
MetaMap-MLP	13.8	30.6	56.4	24.9	47.7	26.2	50.5
CNN	13.5	52.8	68.5	31.8	54	34.8	56
LSTM	13.8	48.1	65.7	31.8	51.9	33.8	54.4
BLSTM	13.8	47.3	66	35.6	57.9	36.9	58.4
DeepTag-M	17.1	53.4	68	37.9	59.9	40.6	61.1
DeepTag	**17.4**	**56.5**	**70.3**	**41.4**	**62.4**	**43.2**	**63.4**

Aggregate prediction performance across the 42 disease codes. We trained a multilayer perceptron (MetaMap-MLP) algorithm and support vector machines (MetaMap-SVM) algorithm on discrete features generated by the MetaMap, which processes a document and extracts medically relevant terms.^25^ CNN refers to a text convolution neural network implementation from Kim.^28^ BLSTM refers to the multitask bidirectional LSTM. DeepTag is our best model, and DeepTag-M is the variation with a meta-disease loss. EM indicates the exact match ratio, which is the percentage of the clinical notes where the algorithm perfectly predicts all of the disease codes. For example, if a note has three true disease codes, then the algorithm achieves an exact match if it predicts exactly these three disease codes, no more and no less. For each precision, recall and *F*_1_ score, there are two ways to compute an algorithm’s performance. First we can take an unweighted average of the score across all the disease codes (unwgt) or we can take an average weighted by the number of test examples in each disease code (wgt). All the algorithms are trained on CSU and tested on a held-out CSU data and the PP data

The bold values indicate the highest value for each evaluation metric

**Fig. 3:**
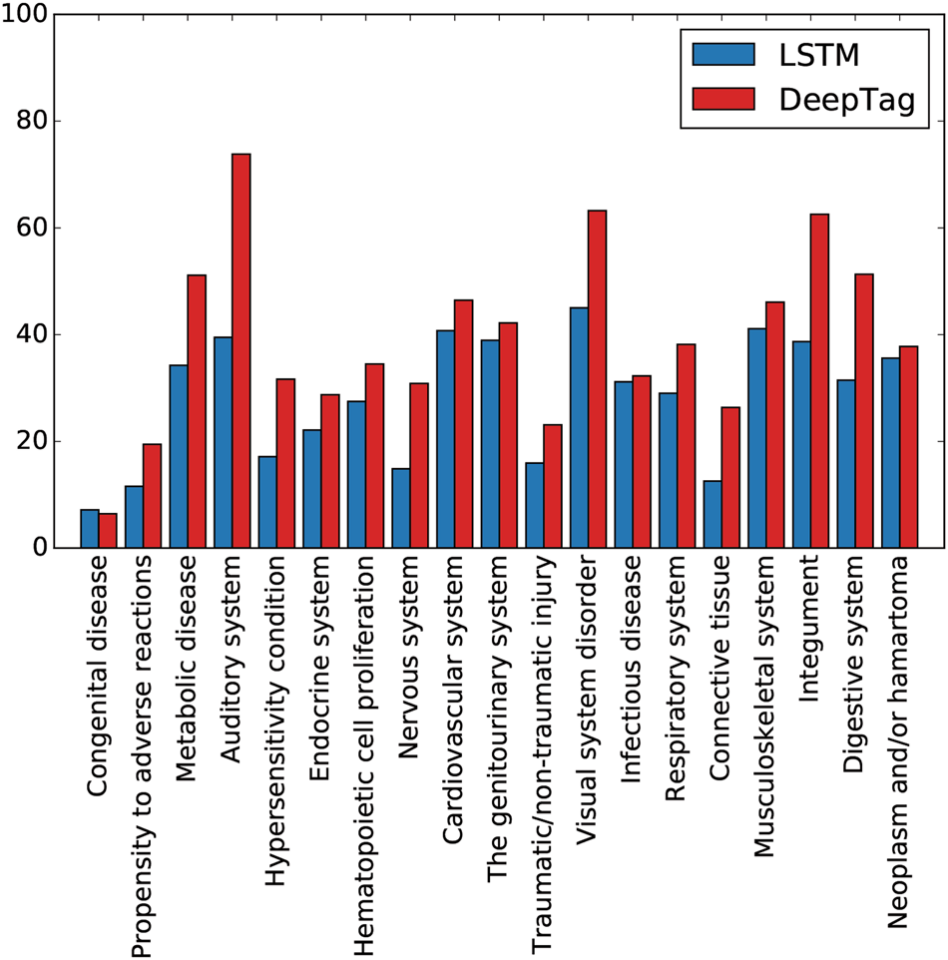
Performance comparison on PP. We compare the per-disease code *F*_1_ score between baseline LSTM model and DeepTag model on the PP dataset. The disease codes are sorted from the least frequent to the most frequent in the training dataset, which comes from CSU

On the CSU dataset, DeepTag and DeepTag-M perform slightly better compared to the baseline deep learning models (CNN, LSTM, and BLSTM). All the deep learning models performed substantially better than MetaMap-SVM and MetaMap-MLP on CSU. DeepTag has higher unweighted precision, recall, and *F*_1_ score compared to the other models, indicating its ability to have good performance on a wide spectrum of diseases. The importance of leveraging similarity is shown on the PP dataset (Fig. 3). Since it is out-of-domain, expert defined disease similarity provide much-needed regularization to make both DeepTag and DeepTag-M out-perform baseline models by a substantial margin, with DeepTag being the overall best model.

### Learning to abstain

Augmenting a tagging system with the ability to abstain (decline to assign codes) can foster human-machine collaboration. When the system does not have enough confidence to make decisions, it has the option to defer to its human counterparts. This aspect is important in DeepTag because after tagging the documents, further analysis from various parties might be conducted on the tagged documents such as investigating the prevalence of certain specific diseases. In order to not mislead further clinical research, having the ability to abstain from making very erroneous predictions and ensuring highly precise tagging is an important feature.

A natural approach to decide whether to abtain on a given document is to check whether the DeepTag prediction confidence—i.e., the output of the final logistic regression—is above a set threshold. We use this approach as the baseline abstention method. This could be suboptimal if DeepTag is over-confident in its predictions. Therefore, we also developed an additional abstention wrapper on top of DeepTag that we call DeepTag-abstain. DeepTag-abstain takes the prediction confidence of the original DeepTag for each of the 42 codes and learns a nonlinear function in order to decide whether to abstain. This learning to abstain approach gives DeepTag-abstain more flexibility to assess the multilabel prediction confidence. See the Supplementary for more details about DeepTag-abstain and the baseline.

In order to evaluate how well DeepTag-abstain performs compared to the baseline, we compute an abstention priority score for each document. A document with higher abstention priority score will be removed earlier than a document with lower score. We then compute the weighted average of *F*_1_ and exact match ratio for all the documents that are not removed.

For both baseline and DeepTag-abstain, we specify a proportion of the documents that need to be removed. We adjust the dropped portion from 0 to 0.9 (dropping 90% of the examples at the high end). An abstention method that can drop more erroneously tagged documents earlier will observe a faster increase in its performance, corresponding to a curve with steeper slope.

**Fig. 4:**
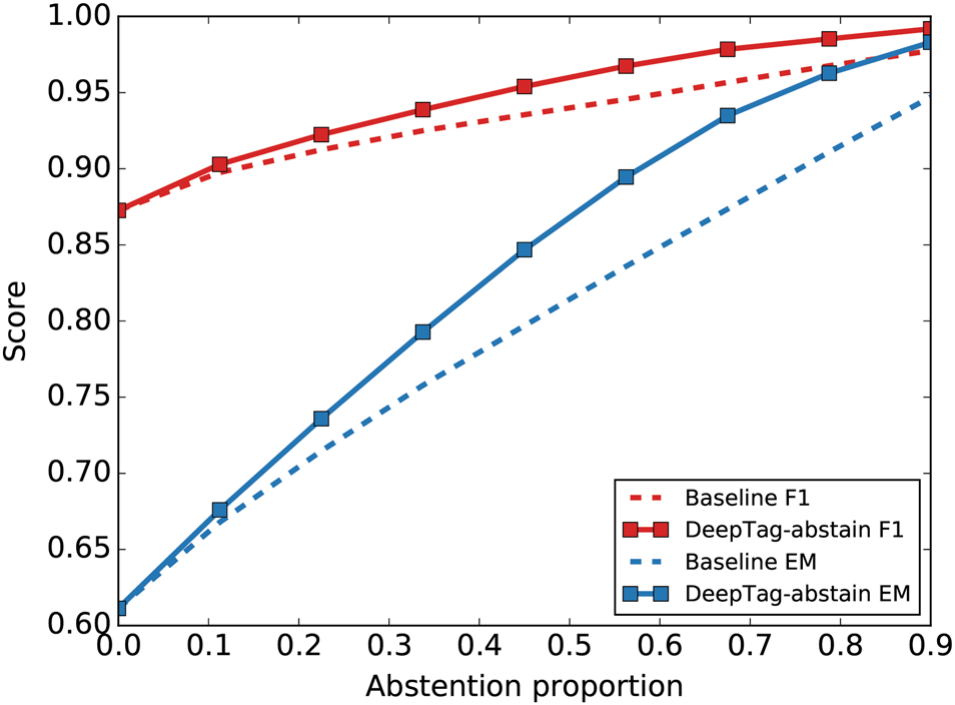
Comparison of the abstention models. DeepTag-abstain is the abstention priority score estimator that uses confidence scores as input and estimate per-document accuracy of a given document. Baseline refers to the abstention scheme where the per-document abstention priority score is computed from individual disease code confidence scores without any learning. As a greater proportion of the examples are abstained from, the performance—*F*_1_ and Exact Match (EM)—of both methods improve. DeepTag-abstain shows faster improvement, indicating that it learns to abstain in more difficult cases

DeepTag-abstain demonstrates a substantial improvement over the baseline in Fig. 4. We note that not all learning to abstain schemes are able to out-perform the baseline. The details of module design and improvement curve for the rest of the modules can be seen in the Supplementary Fig. 2.

## Discussion

In this study, we developed a multilabel classification algorithm for veterinary clinical text, which represents a medical domain with an under-resourced medical coding infrastructure. In order to improve the performance of DeepTag on diseases with rare occurrences, we investigated with loss augmentation strategies that leverage the similarity and dissimilarity between the disease codes. These augmentations provide gains over the LSTM and BLSTM baselines, which are common methods used for these types of prediction tasks. We also experimented with different methodologies to allow the model to learn to abstain on examples where the model is not confident in the predictions. We demonstrate that learned abstention rules out-perform manually set rules.

Our work demonstrates novel methods for applying broad disease codes to clinical records as well as applying those trained algorithms to an external dataset in order to examine cross-hospital generalization. We also demonstrate means to allow human domain experts to use their judgment when automated taggers have a high level of uncertainty in order to improve the overall workflow. We confirm that cross-hospital generalization is a significant concern for learned tagging systems to be deployed in real world implementations that may vary substantially from the data on which they were trained. Even though our work attempts to mitigate this problem, there is significant research to be done to optimize methods for domain adaptation. Our current work is important not only for veterinary medical records, which are rarely coded, but also may have implications for human medical records in countries with limited coding infrastructure and which are important regions of the world for public health surveillance and clinical research.

There are several aspects of the data that may have limited our ability to apply methods from our training set to our external validation set. Private veterinary practices often have data records that closely resemble the PP dataset used to evaluate our methods here. However, the large annotated dataset we used for training is from an academic institution (as these are, largely, the institutions that have dedicated medical coding staff). As can be seen from Table 2, the performance drop due to domain mismatch is non-negligible. The domain shift comes from two parts. First, text style mismatch—private commercial notes use more abbreviations and tend to include many procedural examinations (even though many are non-informative or nondiagnostic).

Second, disease code distribution mismatch—the CSU training dataset focuses largely on neoplasm and several other tumor-related diseases, largely due to the fact that the CSU hospital is a regional tertiary referral center for cancer and cancer represents nearly 30% of the caseload. Other practices will have datasets composed of disease codes that appear with different frequencies, depending on the specializations of that particular practice. A very important path forward is to use learning algorithms that are robust to domain shift, and experimenting with unsupervised representation learning to mitigate the domain shift between academic datasets and private practice datasets.

Currently we are predicting top-level SNOMED-CT disease codes, which are not the SNOMED-CT codes that have been directly annotated on the dataset. Many of the SNOMED-CT codes that are applied to clinical records are coded as “Findings” that are not actual “Disorders” as the actual diagnosis of a patient may not be clear at the time the codes are applied. One example is an animal that is evaluated for “vomiting” and the actual cause is not determined, may have a code of “vomiting(finding)” (300359004) applied and not “vomiting(disorder)” (422400008) and these “non-disorder” disease codes are not evaluated in our current work. However, these are an important subset of codes and represent another means to identify particular patient cohorts with particular clinical signs or presentations, vs. diagnosed disorders.

Another future direction for the abstention branch of this work is to factor human cost and annotation accuracy into the model and only defer when the model believes that human experts will bring improvement to the result within an acceptable amount of cost. This is an interesting direction for experimentation.

## Methods

### Datasets

#### Colorado State University dataset

The CSU dataset contains discharge summaries as well as applied diagnostic codes for clinical patients from the Colorado State University College of Veterinary Medicine and Biomedical Sciences. This institution is a tertiary referral center with an active and nationally recognized cancer center. Because of this, the CSU dataset over-represents cancer-related diseases. Rare diseases in the CSU dataset are diseases like perinatal and mental disorders, but these are also rare in the larger veterinary population as a whole and do not represent a dataset bias. Overall, there are 112,558 unique discharge summaries in CSU dataset. We split this dataset into training, validation, and test set by 0.9/0.05/0.05.

#### Private practice dataset

An external validation dataset was obtained from a regional PP. These records did not have diagnostic codes available and only approximately 3% of these records had a diagnosis placed in the “Problem List” for a particular patient on a particular visit. Out of 352,597 patient records obtained that had Subjective, Objective, Assessment, Plan (SOAP) notes (i.e., notes in the “Subjective, Objective, Assessment, Plan” format), only 13,843 (3.9%) had at least one specific clinical diagnosis listed in the problem list. These diagnoses were free text and not coded to SNOMED-CT by the primary clinicians. Within the SOAP notes, additional diagnoses were frequently found within the “Assessment” field, but were not applied in a consistent standard and by using different levels of evidence, i.e., some of these diagnosis were presumptive, tentative or historical.

Two veterinary domain experts applied SNOMED-CT codes to a subset of these records and achieved consensus on the records used for validation. This dataset (PP) is used for external validation of algorithms developed using the CSU dataset. There are 586 documents in this external validation dataset.

### Data processing

Documents in our corpus have been tagged with SNOMED-CT codes that describe the clinical conditions present at the time of the visit being annotated. Annotations are applied from the SNOMED-CT veterinary extension (SNOMEDCT_VET), which is fully compatible to and is an extension of the International SNOMED-CT edition. It can be accessed in a dedicated browser and is maintained by the Veterinary Terminology Services Laboratory at the Virginia-Maryland Regional College of Veterinary Medicine. Medical coders applying diagnostic codes are either veterinarians or trained medical coders with expertize in the veterinary domain and the SNOMED terminology. The medical coding staff at CSU utilize post-coordinated expressions, where required, for annotations to fully describe a diagnosed condition. For this work, we only considered the core disease codes and not the subsequent modifiers for training our models. The PP dataset was similarly coded using post-coordinated terms following consultation with coding staff at multiple academic institutions that annotate records using SNOMED-CT. We further grouped the 42 disease codes into 18 meta-diseases. More details of this grouping are provided in the Supplementary.

### Difference in data structures

Due to the inherent differences in clinical notes/discharge summaries prepared for patients in an academic setting compared to the shorter “SOAP” format notes prepared in private practice, there are substantial differences in the format as well as the writing style and level of detail between these two datasets. In addition, the private practice records exhibit significant differences in record styles between clinicians, as some clinicians use standardized forms while others use abbreviated clinical notes containing only references to abnormal clinical findings.

As can be seen in Supplementary Fig. 1, both dataset have more than 80% documents associated with more than one disease code, and in terms of document length distribution, PP dataset document is much shorter than CSU dataset, while the average PP document length is 191 words. The average CSU document length is 325 words.

### Algorithm development and analysis

We trained our modeling algorithm on CSU dataset and evaluated on a held-out portion of data from the CSU dataset as well as the PP dataset. We formulated our base model to be a recurrent neural network with LSTM. We additionally decided to run this recurrent neural network on both the forward direction and backward direction of the document (bidrectional), as is found beneficial in Graves et al.^26^ We then built 42 independent binary classifiers to predict the existence of each disease code. This is the architecture found most useful in multilabel classification literature.^21^ The model is trained jointly with binary cross entropy loss. We then augmented this baseline model with two losses: cluster penalty^27^ and a novel meta-disease prediction loss to leverage human expert knowledge in how semantically related these disease codes are.

### Code availability

DeepTag is freely available at https://github.com/windweller/DeepTag.

## Electronic supplementary material

Supplement

## Data Availability

The data that support the findings of this study are available from Colorado State University College of Veterinary Medicine and a private practice veterinary hospital near San Francisco, but restrictions apply to the availability of these data, which were made available to Stanford for the current study, and so are not publicly available. Data are however available from the authors upon reasonable request and with permission of Colorado State University College of Veterinary Medicine and the private hospital.

## Electronic supplementary material

**Supplementary information** accompanies the paper on the *npj Digital Medicine* website (10.1038/s41746-018-0067-8).
