# Supplementary material for: DeepTag: inferring diagnoses from veterinary clinical notes
Source: NPJ Digit Med. 2018 Oct 24;1:60. doi: 10.1038/s41746-018-0067-8 (PMC6550285; doi:10.1038/s41746-018-0067-8)
Supplement: Supplementary file 1 — Supplement [file 41746_2018_67_MOESM1_ESM.pdf]

Supplementary Figures

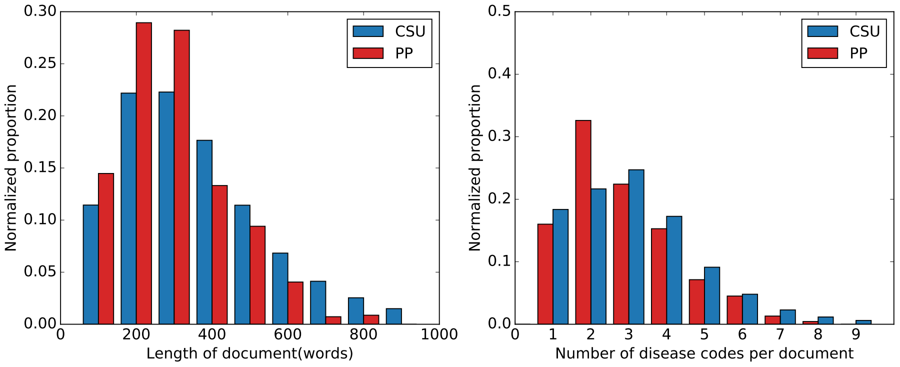

Supplemental Figure 1: **Document length and disease code distribution on CSU and PP dataset.** Proportion of records in each dataset with certain length (number of words) or certain number of disease codes.

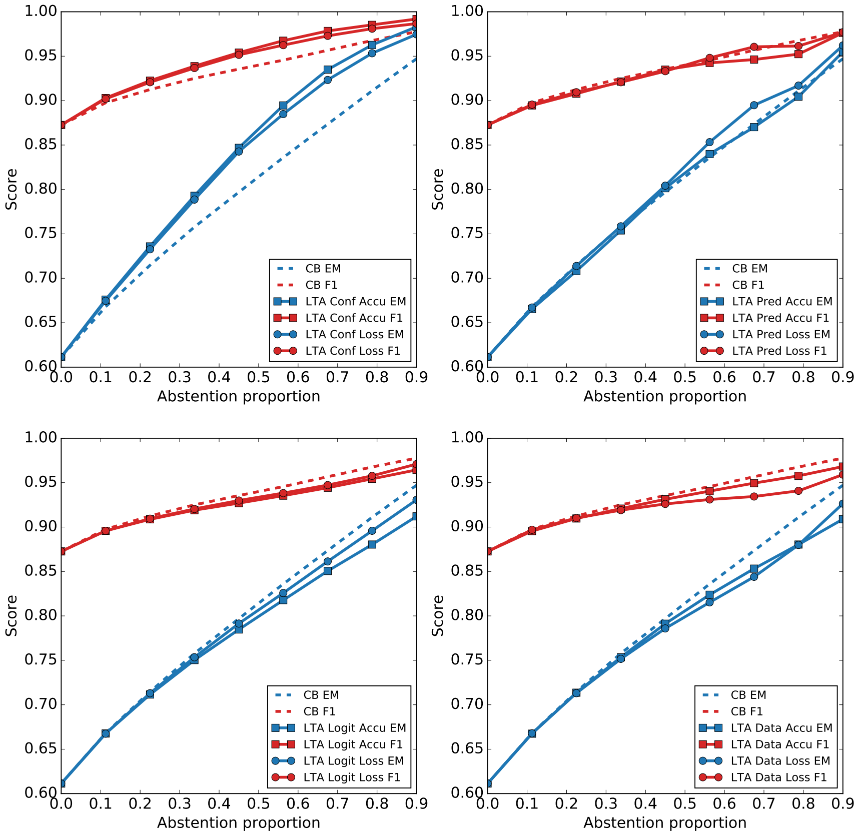

Supplemental Figure 2: **Abstention improvement curve.** Top-left: learning to reject model with confidence score as input, estimate accuracy or loss. Top-right: learning to reject model with post-sigmoid probabilities $\hat{\boldsymbol{y}}$ score as input, estimate accuracy or loss. Bottom-left: learning to reject model with prior-to-sigmoid logits as input, estimate accuracy or loss. Bottom-right: learning to reject model with global max pooled hidden states $\boldsymbol{c}$ as input, estimate accuracy or loss.

Supplementary Materials
DeepTag: inferring diagnoses from veterinary clinical notes

Allen Nie[1,+], Ashley Zehnder[1,+], Rodney L. Page[2], Yuhui Zhang[3], Arturo Lopez Pineda[1], Manuel A. Rivas[1], Carlos D. Bustamante[1,4], and James Zou[1,4,*]

[1]Department of Biomedical Data Science, Stanford University, Stanford, CA 94305, USA
[2]Department of Clinical Sciences, Colorado State University, Fort Collins, CO 80523, USA
[3]Department of Computer Science and Technology, Tsinghua University, Beijing, China
[4]Chan-Zuckerberg Biohub, San Francisco, CA 94158, USA
[*]jamesz@stanford.edu
[+]these authors contributed equally to this work

October 8, 2018

Contents

1 CSU Discharge Summary Format **2**

2 Model Description **2**
2.1 Leveraging disease similarity . . . 3
2.2 Learning to abstain . . . 4
2.3 MetaMap . . . 5
2.4 Text CNN . . . 6

3 Experimental Details **6**
3.1 Main Experiment . . . 6
3.2 Abstention Experiment . . . 6

4 Coding of Private Practice (PP) notes **6**

5 SNOMED Meta-diseases **7**

1 CSU Discharge Summary Format

The Colorado State University discharge summaries contain multiple data fields, including: History, Assessment, Diagnosis, Prognosis, FollowUpPlan, ProceduresAndTreatments, PendingDiagnostics, PendingDiagnosticsComments, Diet, Exercise, DischargeStatus, DischargeDate, Medications, AdditionalInstructions DrugWithdrawal, RecheckVisits, Complications, MedicalComplications, SurgicalComplications and AnesthesiaComplications. We filtered out fields with many null entries as well as the diagnosis related fields, since this is not present in the private practice data. The remaining fields—History, Assessment, Prognosis, DischargeStatus and Medications—were used as the input to train the models.

2 Model Description

We formulate the problem of veterinary disease tagging as a multi-label classification problem. Given a veterinary record $\mathbf{X}$, which contains detailed description of the diagnosis, we try to infer a subset of diseases $\boldsymbol{y} \in \mathcal{Y}$, given a pre-defined set of diseases $\mathcal{Y}$. The problem of inferring a subset of disease codes can be viewed as a series of independent binary prediction problems[12]. The binary classifier learns to predict whether a disease code y_i exists or not for $i = 1, ..., m$, where $m = |\mathcal{Y}|$.

Our learning system has two components: a text processing module and disease code prediction module. Our text processing module uses a long-short-term memory network (LSTM) which has demonstrated their effectiveness in learning implicit language patterns from the text[9]. Our disease code prediction module consists of binary classifiers that are parameterized independently. A long-short-term memory network is a recurrent neural network with a long-short-term memory cell. It takes one word as input, as well as the previous cell and hidden state. Given a sequence of word embeddings $x_1, ..., x_T$, the recurrent computation of LSTM network at a time step t can be described in Eq 1, where σ is the sigmoid function $\sigma = 1/(1+e^{-x})$, and tanh is the hyperbolic tangent function. We use $\odot$ to indicate the hadamard product.

$$
\begin{aligned}
f_t &= \sigma(W_f x_t + V_f h_{t-1} + b_f) \\
i_t &= \sigma(W_i x_t + V_i h_{t-1} + b_i) \\
o_t &= \sigma(W_o x_t + V_o h_{t-1} + b_o) \\
\tilde{c}_t &= \tanh(W_c x_t + V_c h_{t-1} + b_c) \\
c_t &= f_t \odot c_{t-1} + i_t \odot \tilde{c}_t \\
h_t &= o_t \odot \tanh(c_t)
\end{aligned}
\tag{1}
$$

An extension of this recurrent neural network with LSTM cell is to introduce bidirectional passes[4]. Graves et al. shows that introducing bidrectional passes, it can effectively eliminate problems such as retaining long-term dependency when the document is very long. We parameterize two LSTM cells with different set of parameters, one cell is used in forward pass where the sequence is passed in sequentially from the beginning $\{x_1, ..., x_T\}$, one cell is used for backward pass, where the sequence is passed in with reversed ordering $\{x_T, ..., x_1\}$. At the end of both passes, bidirectional LSTM will output two hidden states represents each input x_t, and we stack these two hidden states as our new hidden state for this input $h_t = [\overrightarrow{h_t}; \overleftarrow{h_t}]$.

After computing hidden states over the entire document, we introduce global max pooling over the hidden states, as suggested by Collobert & Weston[2] so that the hidden states will aggregate information from the entire documents. Assuming the dimension of hidden state is d, global max pooling apply an element-wise maximum operation over the temporal dimension of the hidden state matrix, described in Eq 2.

$$
\begin{aligned}
&H = [h_1, ..., h_T],\ H \in \mathbb{R}^{T \times d} \\
&c_j = \max(H_j),\ \text{for } j = 1, ..., d
\end{aligned}
\tag{2}
$$

Then we define a binary classifier for each of the 42 disease code in our pre-defined set. The binary classifier takes in a vector $\boldsymbol{c}$ that represents the veterinary record and outputs a sufficient statistic for the Bernoulli probability distribution indicating the probability of whether a tag should be predicted. For $i = 1, ..., m$:

$$
p(y_i) = \hat{y}_i = \sigma(\theta_i^\intercal \boldsymbol{c}) \tag{3}
$$

We use binary cross entropy loss averaged across all labels as the training loss. Given the binary predictions from the model $\hat{\boldsymbol{y}} \in [0,1]^m$ and correct one-hot label $\boldsymbol{y} \in \{0,1\}^m$, binary cross entropy loss is written as follow. The decision boundary in our model is set to be 0.5.

$$
\mathcal{L}_{\text{BCE}}(\hat{\boldsymbol{y}}, \boldsymbol{y}) = -\frac{1}{m} \sum_{i=1}^{m} y_i \log(\hat{y}_i) + (1 - y_i) \log(1 - \hat{y}_i) \tag{4}
$$

2.1 Leveraging disease similarity

We introduce two penalties that are inspired by the implicit relationships between disease codes that we refer to as meta-diseases. By augmenting our loss with these two penalties, we aim to increase model's ability to predict codes that have fewer instances. We introduce them as DeepTag meta-disease objective and DeepTag-M meta-disease objective.

DeepTag meta-disease objective After defining the meta-diseases for the disease codes, we can use techniques the from multi-task learning literature. Each task corresponds to the binary prediction of one of the 42 disease codes. Jacob et al.[5] proposed a hypothesis that if two tasks are similar, the task-specific parameters for these two tasks—i.e. the corresponding weights in the final neural network layer—should be close in vector space, and vice versa.

We can first compute the mean vector of all disease code embeddings $\bar{\theta} = \frac{1}{m}\sum_{i=1}^{m}\theta_i$. Each disease embedding is a weight parameter θ defined in Eq 3. We can define $\mathcal{J}(k) \subset \{1, ..., m\}$, where $\mathcal{J}(k)$ is a set of disease codes that belong to meta-disease k. Then we can compute a vector for each meta-disease: for $k = 1, ..., K$, $\bar{\theta}_k = \frac{1}{|\mathcal{J}(k)|}\sum_{i\in\mathcal{J}(k)}\theta_i$.

The within-meta-disease closeness constraint Ω_{within} can be computed as the distance between disease code embeddings and the meta-disease vector $\bar{\theta}_k$. Ω_{between} can be computed as the distance between $\bar{\theta}_k$ and $\bar{\theta}$. We formulate this as an additional loss term $\Omega(\Theta)$, and allow three hyperparameter γ_{norm}, γ_{within} and γ_{between} to control the strength of this penalty.

$$\begin{aligned}\Omega_{\text{norm}} &= \sum_{i=1}^{m} ||\theta_i||^2 \\ \Omega_{\text{between}} &= \sum_{k=1}^{K} ||\bar{\theta}_k - \bar{\theta}||^2 \\ \Omega_{\text{within}} &= \sum_{k=1}^{K}\sum_{i\in\mathcal{J}(k)} ||\theta_i - \bar{\theta}_k||^2\end{aligned} \tag{5}$$

DeepTag-M meta-disease objective We propose an additional penalty following the intuition that we want the model to make accurate predictions for the meta-disease even though mistakes can be made on the disease codes. Meta-disease training labels are created by examining whether any of the disease code under this meta-disease has been marked as tagged. Following the same logic, since the disease codes are predicted independently, we can compute the probability of the presence of a meta-disease $\tilde{y}_k$ from the probability of disease codes that belong to this meta-disease.

$$\begin{aligned}p(\tilde{y}_k) &= 1 - \prod_{i\in\mathcal{J}(k)} (1 - p(y_i)) \\ &= 1 - \prod_{i\in\mathcal{J}(k)} (1 - \sigma(\theta_i^{\intercal}\boldsymbol{c}))\end{aligned} \tag{6}$$

After computing the probability of the presence of each meta-disease, given the set of meta-diseases $\tilde{\boldsymbol{y}}$ that are created from our true set of disease codes $\boldsymbol{y}$, we can then compute the binary cross entropy loss between the model's estimation on meta-disease probability and true meta-diseases in Eq 7. We use β to adjust the strength of this penalty.

$$\begin{aligned}\mathcal{L}_{\text{meta}}(p(\tilde{\boldsymbol{y}}), \tilde{\boldsymbol{y}}) = -\frac{1}{K}\sum_{k=1}^{K} & \tilde{y}_k \log(p(\tilde{y}_k)) \\ & +(1 - \tilde{y}_k)\log(1 - p(\tilde{y}_k))\end{aligned} \tag{7}$$

2.2 Learning to abstain

In practice, it is often desirable for the model to forfeit the prediction if the prediction is likely to be incorrect. When the method is used in collaboration with human experts, the model can just defer difficult cases to them, fostering human-computer collaboration. However, this is still an under-explored field in machine learning, and previous research has focused largely on binary-class single-label classification[3]. We formally describe the set-up and our learning-based approach in the following sections, and extend relevant discussion to a multi-label setting.

We propose two abstention settings. Each setting will compute a score α for each document, which we refer to as the abstention priority score. We can then rank these documents using this score α. When user specifies a percentage of documents to be dropped, documents that have high α will be dropped first.

Confidence-based abstention Since our model already outputs a probability for each disease code, if our model is well-calibrated, meaning that the output probability satisfies the following constraint in Eq 8, then our probability should reflect how uncertain the model is about the output.

$$\mathrm{P}_{x,y\sim\mathcal{D}}[y = 1 | f_t(x) = p] = p \;\forall p \in [0, 1] \text{ and } \forall t \tag{8}$$

The notion of calibration means that when the model thinks the chance of a given prediction to be correct is p%, we collect all instances that the model predicts with such probability, and the model in total will be correct p% of the time. A well-calibrated model's output probability corresponds to the model's confidence/certainty on how correct its prediction is. Previous research has shown that binary classifiers with sigmoid scoring function and cross-entropy loss are often well-calibrated[10].

With calibrated $\{p(y_1), ..., p(y_m)\}$, we want to compute how confident the model is on these predictions. For each prediction, the model is more confident if $p(y_i)$ is close to 0 or close to 1. Based on this observation, we can convert the probability into a confidence score with function g: $g(p(y_i)) = \max\{p(y_i), 1 - p(y_i)\}$.

We can now compute the probability of the model getting k disease codes correct on a single example. We choose all subsets from the entire disease code set, and compute the probability of a chosen subset to be correct as well as the probability of the not chosen $(m - k)$ disease codes to be incorrect.

$$\alpha_{\text{conf}} = \sum_{\substack{I \subset \{1,\dots,m\} \\ |I|=k}} \Big(\prod_{i \in I} g(p(y_i))\Big)\Big(\prod_{j \notin I} 1 - g(p(y_j))\Big) \tag{9}$$

The score α_{conf} is an abstention priority score because it is a valid indication of how confident the model's overall output is. We refer to this scheme confidence-based abstention module (or "CB" in Supplemental Figure 2, "Baseline" in main manuscript Figure 4).

Learning-based abstention Instead of computing α from a fixed formula, we can try to link abstention priority score to a value that we care about. For example, we want to drop examples that will induce high loss, or equivalently, examples where predicted result gives a low accuracy. However, we do not have access to ground-truth answers in the real world, instead, we propose that if the data distribution $\mathcal{D}$ between training and deployment are consistent ($x_{\text{test}}, y_{\text{test}} \sim \mathcal{D}$, which is the underlying assumption specified in calibration), then we can learn to estimate loss or accuracy for each example. We can compute a regression target for the learned abstention module using the training dataset's accuracy and loss value for each example (Eq 10), where $d(p) = \mathbf{1}(p > 0.5)$.

$$\begin{aligned} \alpha^i_{\text{accu}} &= \frac{1}{m} \sum_{j=1}^{m} \mathbf{1}[d(\hat{\boldsymbol{y}})^i_j = \boldsymbol{y}^i_j] \\ \alpha^i_{\text{loss}} &= \mathcal{L}_{\text{BCE}}(\hat{\boldsymbol{y}}^i, \boldsymbol{y}^i) \end{aligned} \tag{10}$$

This abstention learning module A can take an input z and output an estimated abstention score $\hat{\alpha}$. We train this module by minimizing minimum square squared error with the regression target:

$$\begin{aligned} \hat{\alpha}^i &= A(z^i) \\ \mathcal{L}_{\text{MSE}} &= \sum_{i=1}^{N} (\alpha^i - \hat{\alpha}^i)^2 \end{aligned} \tag{11}$$

We choose four possible inputs from various parts of the DeepTag model that the DeepTag-abstention module can use to predict accuracy or loss without knowing the ground-truth disease codes. Two choices are obvious: confidence scores $g(\hat{\boldsymbol{y}})$ that is used to compute confidence-based abstention priority score in the previous section, and estimated probability for the presence of each disease code $\hat{\boldsymbol{y}}$, which we have used to compute confidence scores via function $g(\cdot)$. However, since $\hat{\boldsymbol{y}}$ is obtained by applying a sigmoid function to the output of the classifier $\hat{y}_i = \sigma(\theta_i^\intercal \boldsymbol{c})$, then we can also use the prior-to-sigmoid value $\theta_i^\intercal \boldsymbol{c}$ as input. At last, we hypothesize that the representation of document $\boldsymbol{c}$ might also contain relevant information that is useful for model A to determine whether the document is difficult to process.

We fit the model A to estimate α_{learn} in the training set of our data, same split as the one used to train the overall model. We then evaluate on a previously unseen test set.

2.3 MetaMap

MetaMap is a program developed by the National Library of Medicine (NLM)[1]. It processes a document and outputs a list of matched medically-relevant keywords in the given document. We use these keywords as features and map each document into a frequency-encoded bag-of-words vector. The final feature vector size is 57,235. We perform the multi-label classification task with the Multi-layer Perceptron (MLP) and support vector machine (SVM) with linear kernel [1] on these feature vectors.

[1] http://scikit-learn.org

2.4 Text CNN

For the convolutional neural network baseline, we use filter windows of 3, 4, and 5, and each has 340 feature maps. We use rectified linear unit after the convolution, and then apply max pooling over time. We concatenate the the final representations from all filter window sizes, which results in a sentence vector of dimension 1020, comparable to the sentence vector generated by the BLSTM model, which is 1024. The details of our set up follows directly from the implementation of Kim et al.[6].

3 Experimental Details

3.1 Main Experiment

We initialize our model with 100-dimension pretrained GloVE word vectors[11], and we initialize un-matched words in the CSU training data with sampled multivariate normally distributed vectors. We allow all word embeddings to be updated through the training process. We use a recurrent neural network with a 512 dimension LSTM cell, and set the feed-forward dropout rate to be 20%. We use batch size of 32, clipping gradient at 5. We use ADAM[7] optimizer with a learning rate of 0.001.

We trained all models to a maximum of 5 epochs with early stopping, the maximum number of epoch is picked by observing performance on validation dataset. After picking out the best hyper-parameters on validation set, we evaluate all models in-domain generalization performance on the CSU test dataset and out-domain generalization performance on the PP dataset.

After hyperparameter searching, we report models with the hyperparameters that perform well on each dataset. We train each model five times and report the averaged result. For the CSU dataset, we find $\beta = 0.001$ works best for DeepTag-M, and $\gamma_{\text{norm}} = 1e-5, \gamma_{\text{between}} = 1e-4, \gamma_{\text{within}} = 1e-4$ works best for DeepTag. For the PP dataset, we find $\beta = 0.0001$ works the best for DeepTag-M, and $\gamma_{\text{norm}} = 1e-4, \gamma_{\text{between}} = 1e-3, \gamma_{\text{within}} = 1e-3$ works best for DeepTag. We report these results in Table 2 in the main manuscript.

For Table 1 in the main manuscript, we report DeepTag trained with $\gamma_{\text{norm}} = 1e-4, \gamma_{\text{between}} = 1e-3, \gamma_{\text{within}} = 1e-3$ and we regard this as our best setting.

3.2 Abstention Experiment

We use a 3-layer neural network with SELU activation[8] to parameterize abstention model A. The learning to abstain model is trained on various outputs generated by the DeepTag system. All configurations of learning to abstain models are trained optimally for 3 epochs on the training set, and evaluated on the unseen test set.

4 Coding of Private Practice (PP) notes

Our guidelines for applying diagnostic codes to the private practice dataset were derived following consultations and review of coding guidelines from CSU as well consultation with an additional coding professional who helps maintain the SNOMED-veterinary extension and are summarized below:

1. Implied assessments/problems are not be coded unless there is direct evidence to support those diagnoses in the record or noted in the assessment or diagnosis fields. At minimum, diagnoses are applied if there is support from the physical exam and the primary clinician considers it a problem in the patients "Assessment" section in the notes. It is preferred if the assessment/problem is also addressed in the "Plan" section of the note by way of treatments or results from additional diagnostic tests (which are in the "Plan"), but not all diagnoses are addressed here. For example, if the clinician applies a free text diagnosis of obesity, the plan includes weight loss and there is a 7/8 BCS, it is appropriate to code obesity as a problem.

2. Tentative diagnoses are not coded.

3. Historical findings or diagnoses are not coded on a particular visit unless they represent an active problem.

4. Only diagnoses are coded, not signs, symptoms or presenting complaints.

5 SNOMED Meta-diseases

Here we provide the full list of the SNOMED-CT meta-diseases that we used to regularize the training objective of DeepTag. In the list, the numbers correspond to the meta-diseases, and the letters indicate the original SNOMED-CT codes. We manually grouped the 42 SNOMED-CT codes into these 18 meta-diseases, using the analogous grouping of the ICD-9 codes as a guide.

1. Complications of pregnancy, childbirth, and the puerperium
   (a) Disorder of labor / delivery (disorder)
   (b) Disorder of pregnancy (disorder)
2. Diseases of the genitourinary system
   (a) Disorder of the genitourinary system (disorder)
3. Diseases of the musculoskeletal system and connective tissue
   (a) Disorder of connective tissue (disorder)
   (b) Disorder of musculoskeletal system (disorder)
4. Diseases of the skin and subcutaneous tissue
   (a) Angioedema and/or urticaria (disorder)
   (b) Disorder of pigmentation (disorder)
   (c) Disorder of integument (disorder)
5. Certain conditions originating in the perinatal period
   (a) Disorder of fetus or newborn (disorder)
6. Congenital anomalies
   (a) Hereditary disease (disorder)
   (b) Congenital disease (disorder)
7. Injury and poisoning
   (a) Disorder caused by exposure to ionizing radiation (disorder)
   (b) Poisoning (disorder)
   (c) Traumatic AND/OR non-traumatic injury (disorder)
8. Symptoms, signs, and ill-defined conditions
   (a) Hyperproteinemia (disorder)
   (b) Clinical finding (finding)
9. Neoplasms
   (a) Neoplasm and/or hamartoma (disorder)
10. Infectious and parasitic diseases
    (a) Disease caused by Arthropod (disorder)
    (b) Infectious disease (disorder)
    (c) Disease caused by parasite (disorder)
11. Diseases of blood and blood-forming organs
    (a) Anemia (disorder)
    (b) Disorder of cellular component of blood (disorder)
    (c) Disorder of hematopoietic cell proliferation (disorder)
    (d) Disorder of hemostatic system (disorder)
    (e) Spontaneous hemorrhage (disorder)
12. Endocrine, nutritional and metabolic diseses, and immunity disorders
    (a) Autoimmune disease (disorder)
    (b) Disorder of immune function (disorder)
    (c) Hypersensitivity condition (disorder)
    (d) Metabolic disease (disorder)
    (e) Nutritional deficiency associated condition (disorder)
    (f) Nutritional disorder (disorder)
    (g) Obesity (disorder)
    (h) Propensity to adverse reactions (disorder)
    (i) Disorder of endocrine system (disorder)
13. Diseases of the nervous system
    (a) Disorder of nervous system (disorder)
14. Mental disorders
    (a) Mental disorder (disorder)
15. Diseases of the circulatory system
    (a) Disorder of cardiovascular system (disorder)
16. Diseases of sense organs
    (a) Disorder of auditory system (disorder)
    (b) Visual system disorder (disorder)
17. Diseases of the digestive system
    (a) Vomiting (disorder)
    (b) Disorder of digestive system (disorder)
18. Diseases of the respiratory system
    (a) Disorder of respiratory system (disorder)

References

1. Alan R Aronson and François-Michel Lang, *An overview of metamap: historical perspective and recent advances*, Journal of the American Medical Informatics Association **17** (2010), no. 3, 229–236.

2. Ronan Collobert and Jason Weston, *A unified architecture for natural language processing: Deep neural networks with multitask learning*, Proceedings of the 25th international conference on machine learning, 2008, pp. 160–167.

3. Corinna Cortes, Giulia DeSalvo, and Mehryar Mohri, *Learning with rejection*, International conference on algorithmic learning theory, 2016, pp. 67–82.

4. Alex Graves, Santiago Fernández, and Jürgen Schmidhuber, *Bidirectional lstm networks for improved phoneme classification and recognition*, International conference on artificial neural networks, 2005, pp. 799–804.

5. Laurent Jacob, Jean-philippe Vert, and Francis R Bach, *Clustered multi-task learning: A convex formulation*, Advances in neural information processing systems, 2009, pp. 745–752.

6. Yoon Kim, *Convolutional neural networks for sentence classification*, Proceedings of the 2014 conference on empirical methods in natural language processing (emnlp), 2014, pp. 1746–1751.

7. Diederik P Kingma and Jimmy Ba, *Adam: A method for stochastic optimization*, International conference on learning representations, 2015.

8. Günter Klambauer, Thomas Unterthiner, Andreas Mayr, and Sepp Hochreiter, *Self-normalizing neural networks*, Advances in neural information processing systems, 2017, pp. 971–980.

9. Tomáš Mikolov, *Statistical language models based on neural networks*, Presentation at Google, Mountain View, 2nd April (2012).

10. Alexandru Niculescu-Mizil and Rich Caruana, *Predicting good probabilities with supervised learning*, Proceedings of the 22nd international conference on machine learning, 2005, pp. 625–632.

11. Jeffrey Pennington, Richard Socher, and Christopher Manning, *Glove: Global vectors for word representation*, Proceedings of the 2014 conference on empirical methods in natural language processing (emnlp), 2014, pp. 1532–1543.

12. Mohammad S Sorower, *A literature survey on algorithms for multi-label learning*, Oregon State University, Corvallis **18** (2010).
